# Frequency of Cutaneous Disorders in Patients With Celiac Disease

**DOI:** 10.7759/cureus.18148

**Published:** 2021-09-21

**Authors:** Kapeel Dev, FNU Rahul, Kainat Makheja, Jitesh Kumar, Vishal Ahuja, FNU Ekta, Sahrish Dholia, Sidrah Khan, Abdul Subhan Talpur

**Affiliations:** 1 Internal Medicine, Ghulam Muhammad Mahar Medical College, Sukkur, PAK; 2 Internal Medicine, Jinnah Sindh Medical University, Karachi, PAK; 3 Division of Clinical and Translational Research, Larkin Community Hospital, South Miami, USA; 4 Internal Medicine, Chandka Medical College, Karachi, PAK; 5 Internal Medicine, Jinnah Postgraduate Medical Centre, Karachi, PAK; 6 Internal Medicine, Liaquat University of Medical and Health Sciences, Jamshoro, PAK

**Keywords:** celiac disease, autoimmune disease, dermatitis herpetiformis, alopecia areata, psoriasis

## Abstract

Background and objective

Celiac disease is an autoimmune multisystem disorder that is triggered by dietary gluten sensitivity in genetically susceptible individuals. It presents with extraintestinal cutaneous manifestations including dermatitis herpetiformis (DH), atopic dermatitis, psoriasis, urticaria, and alopecia areata. Due to the insufficient availability of data, this study aimed to estimate the frequency of cutaneous manifestation in a Pakistani population with celiac disease.

Methods

This cross-sectional study was conducted from January 2020 to July 2021, and 300 patients with a confirmed diagnosis of celiac disease were enrolled in the study from the internal medicine department of a tertiary care hospital in Pakistan. Celiac disease was confirmed by the presence of immunoglobulin A (IgA) endomysial antibody and IgA tissue transglutaminase antibody. The presence of cutaneous manifestations was assessed with the assistance of a qualified dermatologist and noted in a self-structured questionnaire.

Results

Overall, the most common cutaneous manifestation was DH (16.0%), whereas the second most common cutaneous manifestation was psoriasis (13.8%). DH was most commonly found among males (18.9%), while psoriasis was more common among females (14.12%).

Conclusion

Among the various cutaneous presentations in patients with celiac disease, the most common dermatological manifestation was DH. Therefore, patients with cutaneous manifestations should undergo screening for celiac disease.

## Introduction

Celiac disease is an autoimmune disorder, and it is triggered by dietary gluten sensitivity among genetically susceptible individuals [1]. The gliadin component of gluten peptide provokes an inflammatory T-cell response that leads to the destruction of the villi in the small intestine, leading to malabsorptive symptoms [2]. The classic presentation consists of gastrointestinal symptoms; failure to thrive, diarrhea, bloating, and abdominal pain. However, such classic symptoms are more likely to be seen in patients aged <3 years, while older children and adults present with either extraintestinal manifestations alone or a combination of both [3]. Most of the extraintestinal manifestations result from malabsorption, i.e., iron deficiency anemia, rickets/osteomalacia, and peripheral neuropathy [4].

The wide range of extraintestinal manifestations includes abnormal liver enzymes, stomatitis, myalgias, neuropathy, dermatitis herpetiformis (DH), rashes, alopecia, fatigue, headache, anemia, psychiatric disorders, and infertility. DH is an autoimmune cutaneous response to dietary gluten, which presents as a pruritic papulovesicular rash on the extensor surfaces. A study by Zone concluded that all the patients with DH had associated enteropathy, varying from minimal intestinal abnormality to severe villous blunting, thereby endorsing the idea that it is a pathognomonic feature of celiac disease. The study observed that 60% of patients with DH had silent celiac disease with no intestinal symptoms, whereas 10-20% had classic intestinal symptoms and another 20% had atypical symptoms [5]. Reunala et al. described DH as the most common extraintestinal manifestation of celiac disease [6]. A Finland-based study conducted in 2003 on 18,538 celiac disease patients found that 17% (n=3,121) of the patients had associated DH [6]. Apart from DH, other cutaneous manifestations include atopic dermatitis, psoriasis, urticaria, and alopecia areata [7].

The difference in the prevalence of the condition in different regions can lead to differences in the incidence of cutaneous manifestations in patients with celiac disease. This study aimed to estimate the frequency of cutaneous manifestation in a Pakistani population of patients suffering from celiac disease.

## Materials and methods

This cross-sectional study was conducted from January 2020 to July 2021 in the internal medicine department of a tertiary care hospital in Pakistan. A total of 300 patients with a confirmed diagnosis of celiac disease were enrolled. Participants were enrolled via consecutive convenient non-probability sampling after obtaining informed consent. Ethical review board approval was taken from the Ghulam Muhammad Mahar Medical College IRB before the enrollment of participants (GMMMC/IRB-2020/R-17). Celiac disease was confirmed by the presence of immunoglobulin A (IgA) endomysial antibody and IgA tissue transglutaminase antibody. Participants with hepatitis C, pernicious anemia, diabetes mellitus, systemic lupus erythematosus, and other conditions that commonly have cutaneous manifestations were excluded from the study.

Patients’ gender, age, and time since diagnosis of celiac disease were noted in a self-structured questionnaire. The presence of cutaneous manifestations was assessed with the assistance of a qualified dermatologist and noted in the self-structured questionnaire.

We used SPSS Statistics, version 21.0 (IBM Corporation, Armonk, NY) for data analysis. Continuous variables were tabulated as mean and standard deviation, while categorical data were presented as percentages and frequencies. A p-value of less than 0.05 meant that there was a significant difference in the value between the two groups and the null hypothesis was not valid.

## Results

The mean age of participants was 39 ± 8 years. The majority of participants were male (53.0%), and the mean duration since the diagnosis of celiac disease was 5 ± 2 years (Table 1).

**Table 1:** Demographics of the study participants SD: standard deviation

Demographics	Value
Age (years), mean ± SD	39 ± 8
Gender, n (%)	
Male	159 (53.0%)
Female	141 (47.0%)
Time since the diagnosis of celiac disease (years), mean ± SD	5 ± 2

The cutaneous manifestations were comparable between both genders. However, no statistically significant difference was found between males and females. Overall, the most common cutaneous manifestation was DH (16.0%). It was also the most common manifestation in males. The second most common cutaneous manifestation was psoriasis (13.8%), which was the most common manifestation in females (14.12%) (Table 2).

**Table 2:** Cutaneous manifestations in patients with celiac disease NS: nonsignificant

Lesions	Total, n (%) (n=300)	Male, n (%) (n=159)	Female, n (%) (n=141)	P-value
Dermatitis herpetiformis	48 (16.0%)	30 (18.9%)	18 (12.8%)	NS
Psoriasis	42 (14.0%)	22 (13.8%)	20 (14.2%)	NS
Alopecia areata	18 (6.0%)	10 (6.3%)	8 (5.7%)	NS
Vitiligo	11 (3.6%)	5 (3.1%)	6 (4.3%)	NS
Chronic urticaria	05 (1.6%)	3 (1.9%)	2 (1.4%)	NS
Atopic dermatitis	05 (1.6%)	3 (1.9%)	2 (1.4%)	NS
Vasculitis	02 (0.6%)	1 (0.6%)	1 (0.7%)	NS

## Discussion

The results of our study demonstrated that DH was the most common cutaneous manifestation overall (16.0%) and that too in males. The second most common cutaneous manifestation was psoriasis (13.8%), which was the most common manifestation in females. In 1983, the prevalence of autoimmune skin disease with geographical variations per year was estimated in a study. Males were found to have a comparatively higher prevalence of DH, from ratios ranging from 1.5:1 to 2:1 [8]. Compared to the previous studies, Salmi et al. found a higher prevalence of DH in the Finnish population, between 1980 and 2009 [9].

The human leukocyte antigen (HLA) locus plays a significant role in celiac disease pathogenesis. HLA-DQ2 and -DQ8 prevalence are similar to celiac disease, showing that DH is a clinical manifestation of celiac disease. HLA-DQ2 is expressed in 90% of DH patients. HLA-DQ8 is commonly expressed in patients who lack HLA-DQ2 [10]. All individuals with DH have an accompanying enteropathy that ranges from no atrophy to blunted villi, with full flattening of the villi occurring in 20% of cases. Malabsorption was detected in 10-20% of patients, with unusual symptoms in another 20%, and 60% of patients had “silent” celiac disease [5]. Two individuals with alopecia areata who were endomysial antibody-positive and had celiac disease on small bowel biopsy were described by Fessatou et al. [11]. With the support of a gluten-free diet, both of these patients were able to regrow their hair. However, not all such individuals have had the same outcome [11,12]. Psoriasis is a skin condition caused by T cells. Gluten-free diets have been shown to help patients with psoriasis. Furthermore, patients with positive gliadin antibodies have shown improvement when gluten is restricted. The incidence of positive endomysial and epidermal transglutaminase antibodies in psoriasis patients, however, remains uncertain. Gluten may also be a source of persistent antigen activation in these patients [10,13]. Thyroid problems are three to eight times more common in vitiligo patients. Antithyroid antibodies have been found in many vitiligo patients; the risk of them being elevated in vitiligo patients is five times higher than in the general population [14,15].

The cutaneous manifestation in DH clears rapidly on treatment with dapsone. Dapsone suppresses the inflammation in the skin but has no effect on intestinal abnormality. Despite knowing that gluten is a causative agent, many patients with DH choose not to restrict themselves to a gluten-free diet, which further worsens the disease.

This study has a few limitations. Firstly, since it was a single-center study, the sample size was less diverse and limited. Secondly, due to the cross-sectional nature of the study, a definite association between celiac disease and its dermatological manifestations could not be confirmed. Furthermore, this study also did not establish the impact of cutaneous manifestation on treatment. Therefore, more large-scale studies with diverse sample sizes should be conducted to further explore the association between the two.

## Conclusions

Dermatological manifestation is common in celiac disease. Among the various cutaneous presentations, the most common dermatological manifestation was DH in our cohort. Therefore, patients with cutaneous manifestations like psoriasis, DH, alopecia areata, and others should undergo screening for celiac disease. Furthermore, a close collaborative approach between dermatologists and gastroenterologists is important for the proper recognition of disease presentation. Lastly, patients with DH should follow a gluten-free diet to avoid further worsening of the disease.
